# Influence of Cooperative Learning Intervention on the Intrinsic Motivation of Physical Education Students—A Meta-Analysis within a Limited Range

**DOI:** 10.3390/ijerph18062989

**Published:** 2021-03-14

**Authors:** Taofeng Liu, Mariusz Lipowski

**Affiliations:** 1 Physical Education Institute (Main Campus), Zhengzhou University, Zhengzhou 450001, China; 202033084@sangmyung.kr; 2 Department of Physical Education, Sangmyung University, Seoul 390711, Korea; 3 Faculty of Physical Culture, Gdansk University of Physical Education and Sport, 80-360 Gdansk, Poland

**Keywords:** cooperative learning, intervention effect analysis, physical education students, intrinsic motivation, aggregate effects, heterogeneity

## Abstract

This study was conducted to explore physical education students’ intrinsic motivation and clarify the influence mechanism of cooperative learning methods on learning intrinsic motivation through meta-analysis. In accordance with the Preferred Reporting Items for Systematic Reviews (PRISMA) criteria, we screened literature from the years 2000–2020. The included literature underwent bias analysis on the basis of the five criteria proposed herein. Data were extracted and summarized from the included literature to analyze the causality before and after cooperative learning intervention. Statistical analysis was conducted to determine principal factors affecting physical education students’ learning intrinsic motivation. Simultaneously, the influencing mechanism of cooperative learning on physical education students’ intrinsic motivation was clarified. Results revealed that intrinsic motivation had a high total effect amount. In the experimental group, only three documents determined the significant influence of cooperative learning on physical education students’ intrinsic motivation. Moreover, the time and age differences needed to be considered thoroughly during the intervention. Therefore, cooperative learning intervention can improve physical education students’ intrinsic motivation significantly, and meta-analysis provided a theoretical foundation for applying cooperative learning to the teaching of physical education majors.

## 1. Introduction

Physical education motivation refers to the internal psychological motivation that causes students to frequently participate in physical education learning and exercises [1]. Physical education activity is the result of students’ intrinsic motivation, which determines the tendency, intensity, and persistence of students’ physical education learning and exercising activities [2]. It facilitates the orientation, initiation, adjustment, strengthening, and maintenance of physical education learning and exercise behaviors, showing an important influence on the effect of physical education activities [3]. According to the psychological motivation of students participating in physical education activities, physical education motivation can be divided into internal motivation and external motivation. The former refers to the physical education motivation that arises from the internal psychological factors of students. In other words, students participate in physical education activities entirely out of their needs, desires, and cognition, such as the desire to obtain physical pleasure and psychological pleasure or stimulation from physical education activities, or to satisfy the active, curious, or competitive mentality [4]. The latter refers to physical education activities generated from external factors instead of students themselves; that is, students’ participation in physical education activities is caused by external incentives or pressure, such as wishing to be praised, rewarded, or avoiding punishment [5]. Generally, students’ physical education activities are driven by both internal and external motivations, but at a particular moment, students are often driven by one motivation [6]. Physical education motivation contains differences in the social environment, personality, and individuals. Individual differences are reflected in perception, autonomy, appearance, and value orientation, while differences in the social environment include teaching curriculum, harmony between teachers and students, school facilities, and family support for physical education activities [7].

Perception ability and goal orientation are the fundamental influencing factors of intrinsic motivation, while learning atmosphere and teaching methods will also affect intrinsic motivation. Among them, goal orientation mediates the influence of the learning atmosphere on perception ability and self-determination [8]. Regarding the intrinsic motivation theory, the series of motivation processes proposed by Vallerand are summarized as “social factors→psychological mediator→motivation type→outcome.” To verify the key psychological influencing factors, Ntoumanis found that perception ability was the principal psychological mediator, intrinsic motivation was correlated to positive outcomes, while external regulation and motivation were predictors of negative outcomes [9]. Intrinsic motivation is predominantly affected by perceived usefulness, and usefulness, in turn, is affected by outcome expectation. The expected performance of students participating in school physical education is an essential factor affecting students’ intrinsic motivation [10,11]. Notably, research on the influencing factors of physical education students’ intrinsic motivation is centered on studying perception ability. However, different environments and factors will cause various uncertainties. Therefore, researching and determining factors influencing physical education students’ intrinsic motivation factors are vital for ensuring the teaching of physical education majors.

Cooperative learning is considered a teaching model that can help achieve four learning outcomes: physical, emotional, social, and cognitive. Over the years, cooperative learning intervention has been used in physical education. In the present study, cooperative learning interventions in different educational environments prove that cooperative learning will have a positive effect on motor skills, social skills, cognitive understanding, and the emotion of sports students. In this sense, this teaching model may be a useful teaching strategy to improve intrinsic motivation because it can be associated with the self-determination theory to satisfy students’ basic psychological needs. This model can develop good social relationships between peers and help meet relevant needs [12,13]. It has been analyzed in other school subjects that this model can meet the ability needs by improving students’ physical skills, and thus it has been used in the teaching of a variety of subjects, with the purpose of promoting the unity and cooperation between students. Among them, Dyson found that the cooperative learning program could enable students of all levels to improve their sports, social, and teamwork skills, which is beneficial for both students themselves and others [14]. Cooperative and cognitive learning in physical education learning helps promote students’ participation in physical education activities [15]. In the process of cooperative learning, if coaching time, management time, transition, and waiting time are significantly reduced, physical education exercise expansion and task volume will be significantly increased [16]. Cooperative learning takes study groups as the fundamental teaching organization and systematically utilizes various dynamic factors in teaching, including teachers and teachers, students and students, teachers and students, schools and parents, and schools and society. These factors coordinate with each other to promote the all-round development of group members, thereby achieving the pre-set common teaching goals [17]. Cooperative learning is a teaching strategy based on the overall performance of the study group. Heterogeneous grouping, active mutual dependence, face-to-face facilitating interaction, personal responsibility, social skills and group self-evaluation are the six elements of cooperative learning [18]. In the recent analyses of cooperative learning among physical education majors, most tend to explore the way of cooperative learning, while few have analyzed the influence mechanism of cooperative learning on the intrinsic motivation of physical education students. If the internal mechanism is unclear, it will be a challenge to explore the ways and methods of cooperative learning [19]. Therefore, studying the influences of cooperative learning on intrinsic motivation has become an urgent problem in the teaching of physical education majors.

Regardless of the above problems, previous literature about cooperative learning and physical education’s intrinsic motivation was sorted. In accordance with the Preferred Reporting Items for Systematic Reviews (PRISMA) criteria, we screened literature from the years 2000–2020. On the basis of the research problems proposed herein, we set a fixed retrieval criterion. Data were collected and ordered from all relevant literature. The influence mechanism of cooperative learning intervention on physical education students’ intrinsic motivation was determined through a meta-analysis. The results can provide a theoretical foundation for the teaching of physical education majors.

## 2. Materials and Methods

### 2.1. Literature Retrieval and Selection

Literature published from 2000 to 2020 was retrieved through the Web of Science, Scopus, and EBSCO databases [20]. The keywords included cooperative learning, intervention, physical education students, and intrinsic motivation. Different keyword combinations were utilized: (1) cooperative learning, physical education students, and intrinsic motivation; (2) intervention trial, random trial, physical education students; (3) physical education students, motivation in physical exercise, cooperative intervention. Consequently, a total of 178 documents were retrieved from the above three databases. The date of retrieval was November 2020. Inclusion criteria of documents contain satisfaction of the intervention conditions of cooperative learning, measurement of intrinsic motivation of physical education students, writing in English, and peer review [21].

### 2.2. Literature Screening and Collection

Literature screening was completed following the PRISMA criteria. Data collected included the author(s), publication year, number of samples, and number of interventions [22]. Moreover, the effects of different intervention programs on the data of students’ age, education level, and processing time were analyzed because these factors were mentioned in many documents [23,24,25]. The exact results are presented in Table 1.

### 2.3. Analysis of Comprehensive Effect

The combination of R+Rstudio was utilized. First, all the data were sorted out with the help of Microsoft Excel. Then, they were imported with the import data set of Rstudio [26,27]. Among them, event.e represented the scoring results of physical education students’ intrinsic motivation in the intervention group before the intervention, n.e the scoring results of physical education students’ intrinsic motivation in the intervention group after the intervention, event.c the scoring results of physical education students’ intrinsic motivation in the control group before the intervention, and n.c the scoring results of physical education students’ intrinsic motivation in the control group after intervention. In the perceived locus of causality (PLOC) data used in all the literature [28,29], intrinsic motivation was divided into 5 levels, namely, (1) intrinsic motivation, referring to the interest of physical education; (2) determining motivation, referring to applying these skills to other aspects of life; (3) introducing motivation, referring to the internal driving factors; (4) external changes, referring to that the tasks should be done; and (5) casual motivation, referring to an individual’s capability of attending this physical education class [30,31]. The Likert scale was adopted for scoring [32]. The intrinsic motivation scores of the scale in each document were selected, which were divided into the experimental group (cooperative learning method), the control group (standard learning method), and before and after treatment (measured once before and after teaching).

### 2.4. Bias Analysis

In survey, bias refers to the deviation of all measured values from the actual value, including inaccuracy of the measuring instrument, insufficient sample, unreasonable trial design, unbalanced allocation or grouping, unrandomized sampling, and subjective tendency of the measurer. In research, bias refers to the fact that research results will always deviate more or less from the real situation, which is called error [33,34]. The PEDro scale was used to assess the risk of bias, and the scale could assess the quality of intervention studies, especially randomized controlled trials. The GRADE guide used a four-point scale (high, medium, low, and very low) to assess the data quality [35].

### 2.5. Regression Analysis

For meta-regression analysis, regression analysis was adopted to explore the influence of some experimental characteristics and other covariates on the combined effect in the meta-analysis, thereby clarifying the source of heterogeneity between studies. According to different statistical models, meta-regression analysis can be divided into 2 categories: fixed effect meta-regression analysis and random effect meta-regression analysis [36]. Meta-regression analysis based on the fixed-effect model assumes that multiple studies have a common effect scale. The differences in the effect scales of various studies are mainly caused by random errors. The random effect model assumes that the studies do not have a common effect scale; each study has its effect scale and is defined as a random variable that is normally distributed [37]. The fixed-effect model requires data to satisfy normality, independence between observations, and homogeneity of variance between studies. If data that does not meet these three conditions undergoes a fixed effect analysis, there will be a risk of increasing the error probability. The random effect model only requires the data to meet normality; the other two conditions are not required, and thus the scope of application is expanded [38]. In this meta-analysis, data underwent regression analysis with the regression function metaresult<-metabin in the R package, which mainly analyzed factors such as student age, education level, and processing time.

## 3. Results

### 3.1. Results of Literature Screening

**Figure 1:**
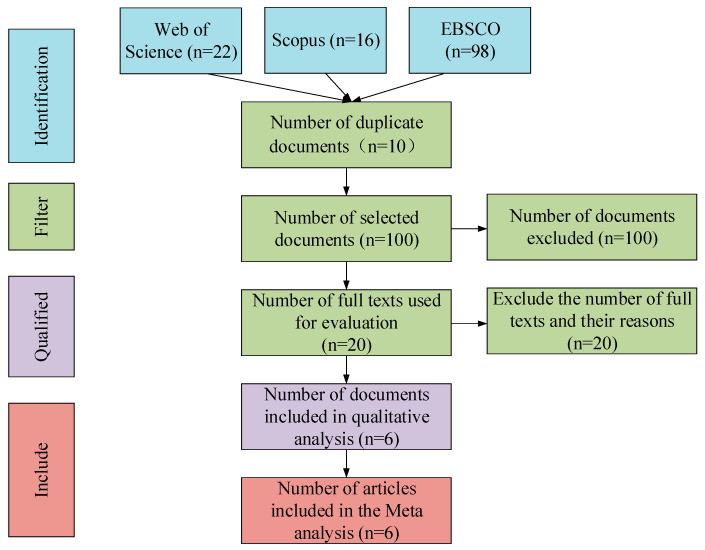
Flowchart of literature screening.

Table 2 lists the retrieval results on the Web of Science, Scopus, and EBSCO databases with different methods used. The numbers of documents retrieved were 22, 16, and 98, respectively. EBSCO includes more libraries, involving papers published in various schools, journals, and periodicals worldwide [39], resulting in more retrieval results. Afterward, the retrieved documents were screened following the PRISMA criteria [40,41], and the results are illustrated in Figure 1. There were 136 documents retrieved in total, while 10 were repeated. According to the selection criteria proposed herein, a large number of documents failed to meet the tests of cooperative learning, physical education major, and intrinsic motivation simultaneously. Hence, 100 documents were excluded. The remaining 26 documents were read intensively, and 20 were found to have incomplete data, making the documents unsuitable for meta-analysis. Consequently, six documents were included for meta-analysis.

### 3.2. Literature Comprehensive Effect

**Figure 2:**
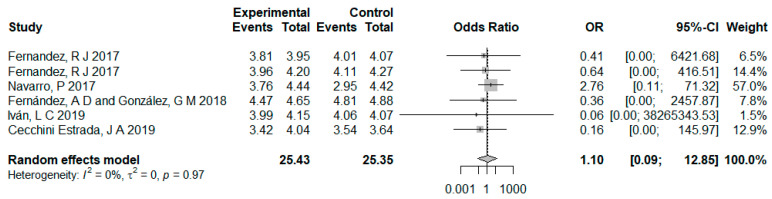
Result analysis of cooperative learning intervention measures influencing physical education students’ intrinsic motivation.

Analyses indicate that the α values of all documents were above 0.7, proving the effectiveness of all data [42]. The research of of Fernández and González [43] and Cecchini Estrada [44] reported the increased intrinsic motivation after cooperative learning intervention; however, no particular *p*-values were given. Regardless of the control group, three studies reported insignificant changes, while the other did not report on this. Figure 2 shows the result analysis of cooperative learning intervention’s influence on the intrinsic motivation of physical education students. The result of the heterogeneity test indicates no statistical significance given that the studies’ variance is tau^2 = 0; tau = 0; I^2 = 0.0% [0.0%; 0.0%]; H = 1.00 [1.00; 1.00] (*p* = 0.9742 > 0.10). Hence, the fixed-effect model can be used for effect amount combination [45], and the result is OR = 1.10, 95% CI: 0.09–12.85, with statistical significance. Notably, the confidence interval of the random effect model was wider than that of the fixed-effect model. The reason was that compared with the fixed-effect model, the random effect model introduced the variance among studies while calculating the standard error.

### 3.3. Results of Bias Analysis

**Figure 3:**
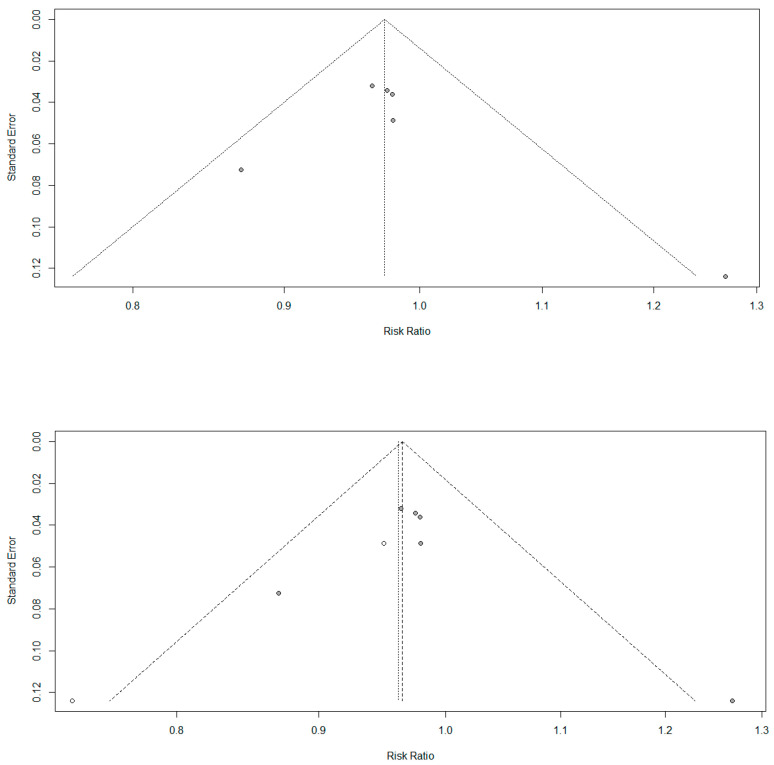
Results of bias analysis.

Figure 3 presents the results of bias analysis. This meta-analysis has the possibility of publication bias. On the right side of the funnel chart, we filled three studies. The heterogeneity test was still not statistically significant; thus, the fixed-effect model was used again, OR = 1.10, 95% CI: 0.09–12.85. Notably, the random effect model had a wider confidence interval than the fixed-effects model, without statistical significance. After correction, the combined effect amount was still statistically significant. Three studies showed a lower risk of bias because they showed a score higher than 0.98. In contrast, the two studies showed a higher risk of bias because they showed a score below 0.98. This was because there were differences in age and education level between different documents, which had a significant reference value for the results of the experiments.

### 3.4. Results of Random Effect Analysis

**Figure 4:**
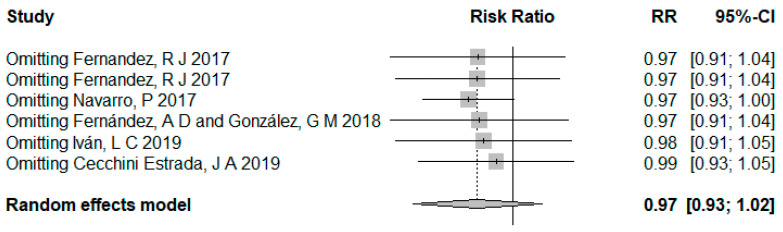
Results of random effect analysis.

Figure 4 shows the results of the random effect analysis. If the fixed effect study was excluded, the combined effect amount of the remaining studies would not be statistically significant, OR = 0.97, 95% CI: 0.93–1.02. The main reason was that the study had the most considerable weight due to the large sample size, and the results were statistically significant. If a statistically significant study with such a considerable weight was excluded, the rest would be small samples of non-statistically significant studies.

### 3.5. Results of Regression Analysis

**Figure 5:**
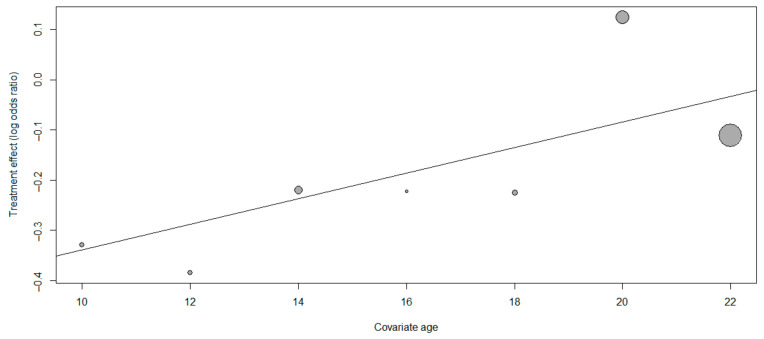
Results of regression analysis.

As shown in Figure 5, different factors (student age, education level, and processing time) were taken as covariance variables. Age in the regression is statistically significant (*p =* 0.0177) [46]. There were no significant differences in education levels and processing time. As age increased, the influence of cooperative learning on the intrinsic motivation of physical education students gradually diminished.

## 4. Discussion

The purpose of this study was to analyze the influence of cooperative learning intervention on the intrinsic motivation of physical education students. According to the research results, we found that the intervention of cooperative learning can effectively improve the intrinsic motivation of physical education students. As shown in Figure 3, three of the six studies supported this conclusion. The other two studies were not included in the analysis due to the bias of the data, which is consistent with the results of the self-determination theory and motivation analysis model. Motivation analysis theory emphasizes how social factors, including cooperative learning, affect different forms of motivation, and this influence is exerted by satisfying basic psychological needs (ability, autonomy, and affinity) [47]. According to the overall effect size, this improvement can be considered small (where d = 0.38, CI is 95% from 0.17 to 0.60, *p* = 0.0004). Due to the low quality of the evidence, there may be some problems with the conclusions, but the results of the current analysis had a good effect on teachers and educators in improving the intrinsic motivation of physical education students [48]. The summarized literature suggests that physical education students have more self-fulfilling needs. Therefore, the main factor affecting the intrinsic motivation of physical education students is self-actualization [49].

The author, publication year, sample size, and intervention number were analyzed. Results suggest that the cooperative intervention processing time was mostly 8–18 h (Table 1), and the form was mostly cooperative games or cooperative training in physical exercise. Among the five interventions based on cooperative learning, we reported four studies with significant improvements in intrinsic motivation. The duration of these effective interventions ranged from three weeks to six months. Iván (2019) reported that after 17 h, the intrinsic motivation of cooperative learning increased from 4.07 to 4.15, an increase of 1.93% [50]. In the study of Fernández-Argüelles and González-González, intrinsic motivation was reduced [51]. Considering that the intervention time of the study was 6 weeks, the duration of the study did not seem to be the most critical factor. However, Figure 3 shows that the three longest studies reported a higher effect size than the other two studies. In general, the longer the processing time, the better the effect on intrinsic motivation. This has been reported in many studies [52,53]. Therefore, according to this result, at least 12 weeks of cooperative learning intervention should be conducted to achieve a significant and relevant improvement in intrinsic motivation.

Another factor that affects the intrinsic motivation of physical education students is age. In the literature, the research objects were divided into two levels: elementary school and high school. According to the obtained results, the increase in intrinsic motivation is higher in elementary school under the condition of the cooperative learning intervention. The results of Navarro (2017) showed that the intrinsic motivation of physical education students after cooperation increased from 3.76 before treatment to 4.44, an increase of 15.3%, and the average treatment of intrinsic motivation in high school was 5% [54,55,56]. Here, the difference in favor of the control group was reported. The samples of the remaining four studies (in which intrinsic motivation has been significantly improved) include elementary school students at an average age of 10.29, junior high school students at an average age of 13.66 and 14.60, and senior high school students at an average age of 20.39. These results are in line with the results reported by Hortigüela-Alcalá et al. Therefore, the largest effect size was observed in the study of Cecchini et al. Overall, the results show that with the increase of participants’ age, the size of the effect will be higher. Therefore, for future cooperative learning interventions with school students, the previous plan to cultivate students’ autonomy should be carried out before the interventions [57].

The results of this systematic review and the differences in the age of participants and the duration of the plan make it impossible to determine whether some plans are more suitable than others. However, the significance of the present study may be to include interventions based on cooperative learning in the physical education teacher training program. In this sense, further research is needed to understand which cooperative learning structure is more effective for improving students’ intrinsic motivation in physical education. In the future, it is recommended to use high-quality interventions as the implementation plan of randomized controlled trials so that more valuable conclusions can be reached [58,59].

## 5. Conclusions

Factors affecting physical education students’ intrinsic motivation and whether cooperative learning can raise students’ intrinsic motivation were analyzed with the meta-analysis, which was achieved through the R language. According to the summary of previous works, cooperative learning intervention is an effective teaching strategy, which can raise the intrinsic motivation of students majoring in physical education. A large amount of literature supports this conclusion. A summary of the literature shows that physical education students are self-actualized, and thus the principal factor affecting physical education students’ intrinsic motivation is self-realization [60,61]. However, student’s age and the processing time should be fully considered in the analysis of physical education students’ cooperative intervention. The intervention effect will be better in primary schools, with a typical processing time of about 6–12 weeks. Such settings can entirely exclude the influences of other factors on the experiment. During the model analysis, no significant differences were found in the results of the fixed-effect model, while the random effect model was different, which was due to the differences in age and processing time [62]. Although the influences of cooperative learning on physical education students’ intrinsic motivation were analyzed, the following limitations were found. First, only documents in English were included, leading to a small number of documents analyzed. In addition, the inclusion criteria should be relatively strict, and most of the included documents were peer-reviewed. Master and doctoral dissertations are relatively complete and sufficient, which can make the analysis more accurate. Nevertheless, these dissertations were excluded in this study. Second, the fixed-effect model was utilized for analysis [63]. Due to the use of scoring results, the difference between intervention effects was minimal, which required a large amount of data and diversified data processing methods. Here, only simple bias and regression analyses were adopted in this study, which could not analyze how cooperative learning affected the intrinsic motivation of physical education students. Therefore, these two aspects need to be analyzed profoundly in the future, so as to continuously improve the proposed theory.

## Figures and Tables

**Table 1:** Collection of literature data.

First Author	Year	Number of Samples	Academic Stage	Distribution Principle	Intervention Group	Control Group	Generation	Processing Time
Fernandez, R. J.	2017	217	High school	Randomly assigned	104	113	12-17	8–12 h
Fernandez, R. J.	2017	249	High school	Randomly assigned	112	137	13-16	8 h
Navarro, P.	2017	104	Primary school	fixed allocation	50	54	10-12	8 h
Fernández, A. D., and González, G. M.	2018	47	Primary school	fixed allocation	31	16	10-12	9 h
Iván, L. C.	2019	61	High school	Randomly assigned	29	32	13-17	17 h
Cecchini Estrada, J. A.	2019	372	High school	Fixed allocation	190	182	12-17	6 months

**Table 2:** Literature summary analysis.

Database	Search Method-1	Search Method-2	Search Method-3	Total
Web of Science	9	4	9	22
Scopus	2	10	4	16
EBSCO	40	18	40	98
Total	51	32	53	136

## Data Availability

The raw data supporting the conclusions of this article will be made available by the authors, without undue reservation.
